# More evidence of the health risks of normal weight obesity: the association with systemic inflammation

**DOI:** 10.3389/fmed.2025.1695935

**Published:** 2025-11-18

**Authors:** Rachel Liu-Galvin, Frank A. Orlando, Aaron A. Saguil, Ara Jo, Kristy B. Smith, Andrew M. Miller, Danielle S. Nelson, Elizabeth C. Sanders, Arch G. Mainous

**Affiliations:** 1 Department of Health Services Research, Management and Policy, University of Florida, Gainesville, FL, United States; 2 Department of Community Health and Family Medicine, University of Florida, Gainesville, FL, United States

**Keywords:** body composition, body fat percentage, body mass index, inflammation, CRP - C-reactive protein, screening, cardiometabolic health, normal weight obesity

## Abstract

**Background:**

Normal weight obesity (NWO) – a normal body mass index (BMI) with high body fat percentage (BF%) – has been linked to increased cardiometabolic risk. This study examined whether NWO is associated with systemic inflammation.

**Methods:**

Using 2017–2018 NHANES data, we categorized adult respondents aged 18–59 with BMI ≥ 18.5 into four groups:

1. Reference: Normal BMI (18.5–24.9) with normal BF% (< 25% males/ < 35% females)
2. NWO: Normal BMI with high BF% (≥ 25% males/ ≥ 35% females)
3. Elevated BMI (≥ 25) with normal BF%
4. Elevated BMI with high BF%

Survey-weighted logistic regression examined associations with elevated hs-CRP (> 3.0 mg/L), adjusting for age and race/ethnicity. Sex-stratified analyses were also conducted.

**Results:**

Inflammation prevalence was 32.7% overall, highest among individuals with elevated BMI and high BF% (43.6%). Compared to the reference group, individuals with NWO had over 3-fold increased odds of inflammation [AOR 3.34 (95% CI: 1.83, 6.08)]; individuals with elevated BMI and high BF% had over 6-fold increased odds [AOR 6.19 (95% CI: 3.66, 10.50)]. Elevated BMI with normal BF% was not significantly associated with inflammation.

In sex-stratified analyses, NWO was associated with inflammation in both males [AOR 4.44 (95% CI: 1.62, 12.10)] and females [AOR 2.78 (95% CI: 1.40, 5.52)]. Elevated BMI and high BF% was also associated with inflammation in both sexes.

**Conclusion:**

In this cross-sectional study, NWO was associated with inflammation, although causality cannot be inferred. Reliance on BMI alone may misclassify cardiometabolic risk therefore BF% should be considered in clinical assessments.

## Introduction

Chronic low-grade inflammation has been linked to the development and progression of cardiometabolic diseases, including hypertension, atherosclerosis, coronary artery disease, and type 2 diabetes, as well as cancer (1–15). High-sensitivity C-reactive protein (hs-CRP) is a well-established blood test used to measure inflammation, and elevated hs-CRP levels have been associated with an increased risk of future cardiovascular events, as well as all-cause, cardiovascular-related, and cancer-related mortality (16–21).

Obesity is a driver of both inflammation and chronic and cardiometabolic conditions (22, 23), and a growing body of evidence suggests that assessing body fat percentage (BF%), rather than relying solely on body mass index (BMI), may be more relevant in determining health risks and mortality (24, 25). Although the gold standard definition of obesity is an excess of body fat (26), BMI is the most commonly used metric in clinical practice. Thresholds for overweight and obesity are defined by BMI, and current guidelines rely on BMI thresholds to determine screening and interventions for a variety of weight-related comorbidities, such as prediabetes and type 2 diabetes (27). The USPSTF also recommends clinicians refer adults with a BMI of 30 or higher to intensive, multicomponent behavioral interventions (28).

While BMI has long been the primary metric in clinical weight management, it is a poor surrogate marker of actual adiposity or body fat percentage (29). Approximately 30 million Americans have a high BF% despite having a normal BMI (30). This condition, referred to as normal weight obesity (NWO) (31) is associated with significant health risks. Goodpaster et al. found that greater visceral fat among normal weight males was associated with more than double the odds of metabolic syndrome (24). More recent studies have shown that a significant proportion of individuals with NWO have prediabetes, undiagnosed diabetes, hypertension, or metabolic dysfunction-associated steatotic liver disease (32–34). Lower lean body mass in relation to body fat is being increasingly recognized as a critical contributor to cardiometabolic risk, more so than obesity itself (35–37). Thus, assessing patients based solely on BMI may misclassify a substantial number of those who are at high risk (25, 38).

Incorporating BF% measurements may provide additional, important insights into the risk of inflammation and related cardiometabolic risk compared to relying on BMI alone. Understanding the association between different body composition profiles and inflammation may reveal important limitations in the current reliance on BMI-based classifications of obesity and inform more targeted screening and prevention strategies. This study examined whether having NWO is associated with elevated inflammation in a nationally representative sample of U.S. adults from the National Health and Nutrition Examination Survey (NHANES).

## Methods

### Data source

We conducted a cross-sectional analysis using data from the 2017 to 2018 NHANES, a large, nationally representative survey of the non-institutionalized U.S. population administered by the Centers for Disease Control and Prevention (CDC) (39). More information about the NHANES methodology and protocols is available on the CDC website (40). NHANES participants answer questions about their health and undergo a standardized medical examination including blood tests for biomarkers and body measurements. The 2017–2018 cycle was selected as the most recent cycle to include measurements from whole-body dual-energy X-ray absorptiometry (DXA). This study was conducted using publicly available and deidentified data. As such, it did not involve human subjects research and did not require Institutional Review Board review. The study adheres to the Strengthening the Reporting of Observational Studies in Epidemiology (STROBE) guidelines for reporting observational research (41).

### Study population

The study population consisted of adult respondents from the 2017 to 2018 NHANES who were eligible for DXA, which was conducted only among individuals aged 18–59 years. NHANES further restricted DXA eligibility to non-pregnant participants with no radiographic contrast use in the past 7 days, and with self-reported weight ≤ 450 pounds and height ≤ 6 feet 5 inches (42).

**FIGURE 1:**
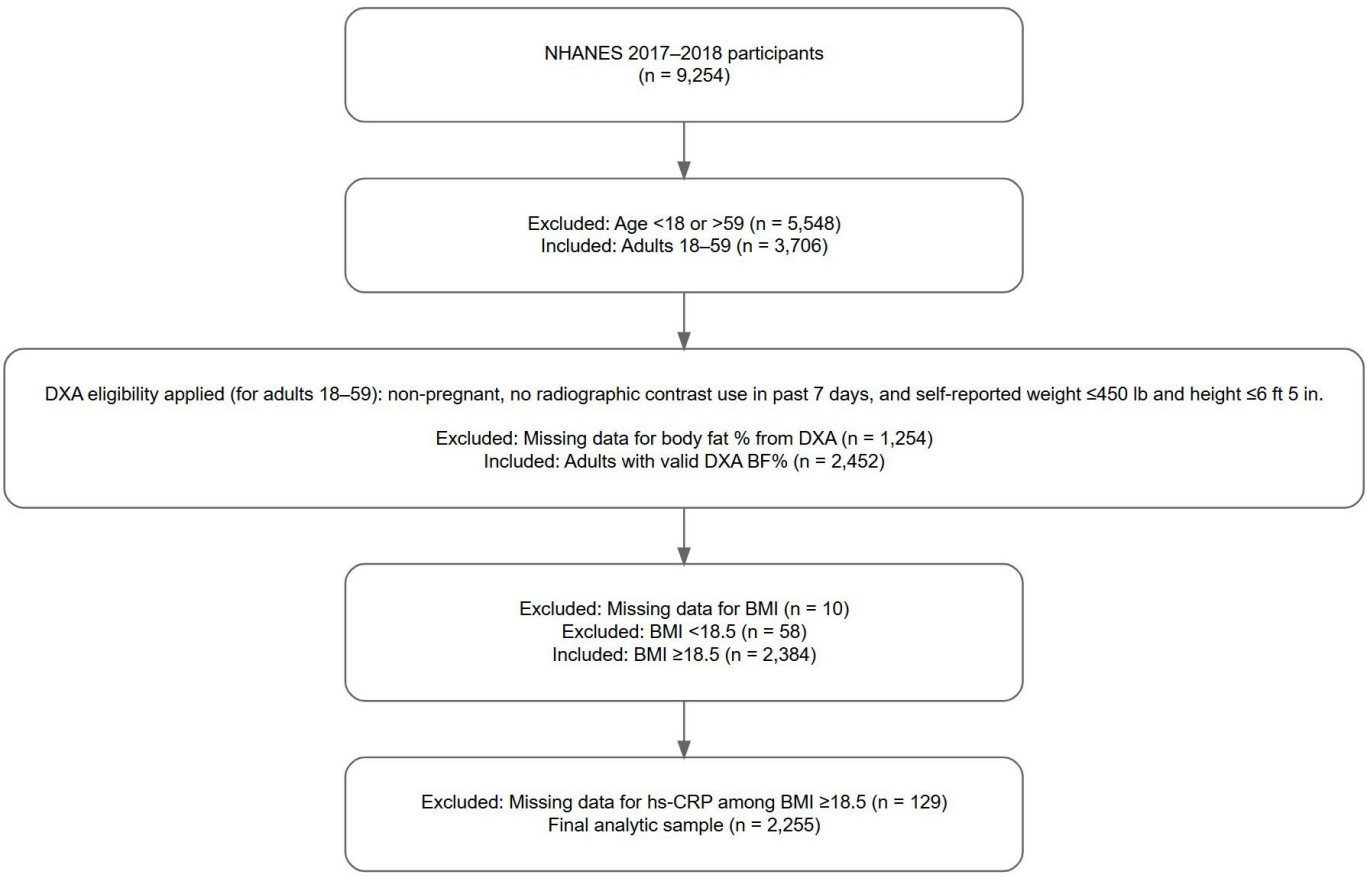
Selection procedure for the study population. Participants were drawn from the 2017 to 2018 National Health and Nutrition Examination Survey (NHANES). Inclusion criteria required participants to have non-missing data for body mass index (BMI), body fat percentage as measured by whole body dual-energy X-ray absorptiometry (DXA), and high-sensitivity C-reactive protein (hs-CRP), with BMI ≥ 18.5. NHANES DXA eligibility criteria included non-pregnant participants aged 18–59 years, with no radiographic contrast use in the past 7 days, and self-reported weight ≤ 450 pounds and height ≤ 6 feet 5 inches. The final unweighted sample size was 2,255 individuals, representing a weighted population of 114,132,307 U.S. adults.

Individuals with BMI < 18.5 were excluded to reduce potential confounding from undernutrition and to align with the study’s aim to assess inflammation risk across different body composition groups in those with clinically defined healthy or elevated BMI, with the term “elevated BMI” used in this study to refer to BMI ≥ 25. Participants were included if they had non-missing data for BMI, total body BF%, and high-sensitivity C-reactive protein (hs-CRP). Figure 1 (flow diagram) depicts the study population selection. The final unweighted sample size was 2,255 individuals, representing a weighted population of 114,132,307 U.S. adults.

### Study outcome

The primary outcome measure of this study was the presence of systemic inflammation, defined as hs-CRP levels greater than 3.0 mg/L. This threshold corresponds to the high-risk category for major coronary events as defined by the American Heart Association, which classifies hs-CRP levels into three categories: low risk (< 1.0 mg/L), average risk (1.0–3.0 mg/L), and high risk (> 3.0 mg/L) (19). These cut-points represent approximate tertiles of the distribution of hs-CRP levels in the adult population, with the high-risk category associated with an approximately 2-fold increase in the relative risk of major coronary events compared to the low-risk category (19). The threshold of 3.0 mg/L is also consistent with that used in previous studies (16, 20, 43–48).

### Independent variable

We assessed the association between body composition group, defined by categories of BMI and BF%, and the odds of elevated inflammation. Individuals with a normal BMI and a normal BF% served as the reference group. BF% was measured using whole body dual-energy X-ray absorptiometry (DXA), the gold standard for assessing body composition (49, 50).

Normal BMI was defined as 18.5–24.9 kg/m^2^ and elevated BMI as ≥ 25 kg/m^2^. Normal BF% was defined as < 25% in males and < 35% in females, and high BF% as ≥ 25% in males and ≥ 35% in females. The thresholds for BF% were selected based on the American Association of Clinical Endocrinologists/American College of Endocrinology Obesity Task Force 1998 position statement on the prevention, diagnosis, and treatment of obesity, and those used in prior studies (51–53). Individuals were categorized into four mutually exclusive groups:

1. Reference Group: Normal BMI with a normal BF%.
2. Group with Normal Weight Obesity: Normal BMI with a high BF%.
3. Elevated BMI with a normal BF%.
4. Elevated BMI with a high BF%.

### Statistical analysis

We conducted survey-weighted analyses accounting for the NHANES complex sampling design, using the appropriate weight, strata, and cluster variables for our study population. Descriptive statistics were calculated to compare participant characteristics across body composition groups. Modified Rao-Scott Chi-Square tests of independence, with variance estimated using Taylor Series Linearization, were used to assess differences between these groups.

Unadjusted and adjusted survey-weighted logistic regression models were fitted to examine the association between body composition group and the odds of elevated hs-CRP (> 3.0 mg/L). Models were fitted for the overall study population, as well as separately for males and females, to account for sex-based differences in body composition (54). It should be noted that some sex-stratified subgroups, such as males with normal weight obesity and females with elevated BMI and normal BF%, had small unweighted sample sizes; therefore, the precision of these estimates (as reflected in their confidence intervals) should be interpreted with caution. The adjusted models controlled for age and race/ethnicity. Given our aim was to compare how different body composition profiles defined by BMI and BF% thresholds are associated with inflammation in a manner that reflects clinical practice, where classification based on these thresholds does not vary according to comorbidities, we limited adjustment to key demographic variables (age and race/ethnicity), which were treated as background confounders. Because our objective was to evaluate potential misclassification of inflammatory risk when relying on BMI alone, rather than to estimate the independent association of NWO with inflammation, other factors such as lifestyle behaviors and comorbidities were not adjusted for, as these may act as mediators on the pathway between body composition and inflammation. Adjusting for them could constitute overadjustment and obscure the associations we aimed to describe between different body composition profiles and inflammation in the general adult U.S. population.

As a sensitivity analysis, we also fitted all models again after excluding individuals with hs-CRP levels > 10 mg/L (*n* = 153) to account for the potential influence of acute inflammation that may have led to substantially elevated hs-CRP unrelated to adiposity. The results of this sensitivity analysis are presented in Supplementary Appendix Table 1 and were compared to the main analyses to assess the robustness of the findings.

All analyses were conducted using R version 4.4.1 (2024-06-14, ucrt) and RStudio 2024.12.1 (Posit Software, PBC) (55, 56). Survey-weighted logistic regression models were fitted using the survey package with svydesign() and svy(glm) to incorporate the NHANES strata, cluster, and weight variables, providing nationally representative estimates for the U.S population. All *p*-values were two-sided, with *p* < 0.05 considered statistically significant. Due to the use of design-adjusted degrees of freedom in survey-weighted analyses, *p*-values are based on the t-distribution and may differ slightly from confidence intervals derived using normal (z) approximations. This may result in marginal discrepancies between *p*-values and 95% CIs.

## Results

The study sample included 2,255 (unweighted) individuals, representing 114,132,307 (weighted) non-institutionalized U.S. adults, of whom 1,077 (weighted, 56,004,781) were male and 1,178 (weighted, 58,127,527) were female. Table 1 presents the distribution of participant characteristics across the four body composition groups. Overall, 9.2% of the population had NWO, representing 30.5% of all individuals classified as having a normal BMI.

**TABLE 1:** Distribution and characteristics of U.S. adults aged 18–59 years by body composition group based on body mass index (BMI) and body fat percentage, National Health and Nutrition Examination Survey (NHANES) 2017–2018.

Category	Normal BMI with normal BF%	Normal BMI with high BF% (normal weight obesity)	Elevated BMI with normal BF%	Elevated BMI with high BF%	*P*-value
**Participant distribution**					
Unweighted sample size	455	216	188	1,396	–
Weighted sample size	23,842,161	10,472,061	10,053,780	69,764,306	–
Weighted prevalence in the overall study population (%)	20.9	9.2	8.8	61.1	–
Weighted prevalence (%) within each BMI category	69.5	30.5	12.6	87.4	–
**Participant characteristics**					
Elevated hs-CRP (%)	10.9	29.2	12.0	43.6	< 0.0001
Age (%)					0.0510
18–34 years	57.7	37.6	48.8	35.5	–
35–49 years	24.4	30.2	32.0	38.3	–
50 and above years	17.9	32.2	19.3	26.1	–
Sex (%)					< 0.0001
Male	43.6	33.9	79.3	48.9	–
Female	56.4	66.1	20.7	51.1	–
Race (%)					0.0006
Non-Hispanic White	65.9	64.9	53.4	55.0	–
Non-Hispanic Black	10.2	5.2	13.3	9.8	–
Hispanic	11.7	13.3	25.5	23.8	–
Other	12.1	16.6	7.8	11.4	–

All percentages are weighted to represent the U.S. non-institutionalized adult population. Individuals with BMI < 18.5 kg/m^2^ were excluded.

The weighted prevalence of elevated hs-CRP (> 3.0 mg/L) was 32.7% in the overall study population, 24.7% in males, and 40.3% in females. The weighted prevalence of elevated hs-CRP was highest among individuals classified as having an elevated BMI with a high BF%, with 43.6% of this group classified as having elevated hs-CRP. This was followed by individuals classified as having normal weight obesity, among whom 29.2% had elevated hs-CRP levels.

There were no statistically significant differences in age distribution across the groups (Table 1). However, significant differences were observed in sex and race/ethnicity. A greater proportion of individuals with an elevated BMI and a normal BF% were male, while a greater proportion of individuals with NWO were female. Non-Hispanic White individuals were more prevalent in both groups with a normal BMI, including the group with NWO, whereas Hispanic individuals were more prevalent in both groups with elevated BMI.

The results of survey-weighted logistic regression presented in Table 2 show that, among the overall study population, and after adjusting for age and race/ethnicity, individuals with NWO had more than 3-fold increased odds of inflammation compared to the reference group (individuals with a normal BMI and a normal BF%), with an adjusted odds ratio (AOR) of 3.34 (95% CI: 1.83, 6.08). Individuals with an elevated BMI and a high BF% had more than 6-fold increased odds (AOR 6.19; 95% CI: 3.66, 10.50), while individuals with an elevated BMI and a normal BF% did not have significantly increased odds of inflammation compared to the reference group.

**TABLE 2:** Association between body composition and odds of elevated high-sensitivity C-reactive protein (hs-CRP) levels among U.S. adults aged 18–59 years: unadjusted and adjusted odds ratios from survey-weighted logistic regression, overall and stratified by sex, National Health and Nutrition Examination Survey (NHANES) 2017–2018.

Body composition group	Unadjusted OR (95% CI)	*P*-value	Adjusted OR* (95% CI)	*P*-value
**Overall study population**				
Reference group: normal BMI with normal BF%	1.00	–	1.00	–
Normal BMI with high BF% (normal weight obesity)	3.38 (1.87, 6.11)	0.0017	3.34 (1.83, 6.08)	0.0056
Elevated BMI with normal BF%	1.11 (0.64, 1.92)	0.7191	1.09 (0.63, 1.90)	0.2246
Elevated BMI with high BF%	6.32 (3.84, 10.40)	< 0.0001	6.19 (3.66, 10.50)	< 0.0001
**Males**				
Reference group: normal BMI with normal BF%	1.00	–	1.00	–
Normal BMI with high BF% (NWO)	4.67 (1.62, 13.4)	0.0144	4.44 (1.62, 12.10)	0.0228
Elevated BMI with normal BF%	1.00 (0.40, 2.51)	0.9945	0.957 (0.401, 2.28)	0.9230
Elevated BMI with high BF%	4.71 (2.46, 9.01)	0.0005	4.39 (2.28, 8.43)	0.0030
**Females**				
Reference group: normal BMI with normal BF%	1.00	–	1.00	–
Normal BMI with high BF% (NWO)	2.76 (1.43, 5.36)	0.0109	2.78 (1.40, 5.52)	0.0221
Elevated BMI with normal BF%	2.07 (1.10, 3.93)	0.0449	2.05 (1.09, 3.86)	0.0602
Elevated BMI with high BF%	8.57 (4.85, 15.1)	< 0.0001	8.51 (4.70, 15.40)	0.0002

*Models adjusted for age and race/ethnicity. *P*-values are based on design-adjusted t-distributions; minor differences from 95% CIs reflect survey-weighted estimation. Some sex-stratified subgroups [e.g., males with normal weight obesity and females with elevated body mass index (BMI) and normal body fat percentage (BF%)] had small unweighted sample sizes; therefore, the precision of these estimates (as reflected in their confidence intervals) should be interpreted with caution. Individuals with BMI < 18.5 kg/m^2^ were excluded.

Also shown in Table 2 are the results of the sex-stratified analyses. In the sex-stratified models which adjusted for age and race/ethnicity, compared to the reference group, having NWO was associated with inflammation in both sexes, with a stronger association in males (AOR 4.44; 95% CI: 1.62, 12.10) than in females (AOR 2.78; 95% CI: 1.40, 5.52). Having an elevated BMI with a high BF% was also associated with inflammation in both sexes, and the association was stronger in females (AOR 8.51; 95% CI: 4.70, 15.40) than in males (AOR 4.39; 95% CI: 2.28, 8.43). In contrast, having an elevated BMI with a normal BF% was not significantly associated with inflammation in males, females, or in the overall study population.

The results of the sensitivity analysis, in which all models were refitted after excluding individuals with hs-CRP levels > 10 mg/L (*n* = 153) to account for the potential influence of acute inflammation, confirm the robustness of the main findings, showing no meaningful differences in the magnitude or direction of odds ratios or in the statistical significance of the associations across the overall and sex-specific models.

## Discussion

This nationally representative analysis of U.S. adults reveals a clear link between systemic inflammation and a high BF%, even among individuals with a normal BMI. In the overall study population, individuals with NWO had more than three times higher odds of inflammation compared to those with a normal BMI and a normal BF%, even after adjusting for age and race/ethnicity. Among individuals with a normal BF%, there was no significant difference in odds of inflammation between those with a normal BMI and those with an elevated BMI. Notably, nearly one-third (30.5%) of individuals with a normal BMI had NWO, representing 9.2% of the overall study population. These findings add to evidence that relying on BMI alone may misclassify cardiometabolic risk for a significant proportion of the population by overlooking individuals with excess body fat despite a normal BMI (25, 38).

When stratified by sex and adjusted for age and race/ethnicity, both males with NWO and males with an elevated BMI and a high BF% had similarly increased odds of inflammation compared with the reference group (males with a normal BMI and a normal BF%). Meanwhile, the increased odds of inflammation among females with an elevated BMI and a high BF% were markedly higher than they were for males with this body composition profile, when comparing both with their respective reference groups. A possible hypothesis is that once BMI and BF% exceed a certain threshold there could be a synergistic effect that is more deleterious for females than for males, although this cannot be confirmed given the cross-sectional nature of the study. It may also suggest that any lower propensity for inflammation observed among females at a normal BMI could be lost once the elevated BMI threshold is surpassed. One possible explanation for these findings is sex-specific variation in the association of inflammation with excess adiposity. A prior study that examined the association between BMI and hs-CRP in 119 adults observed that the pro-inflammatory effect associated with increases in BMI was greater in females than in males (57). In other studies that looked specifically at body fat, Cartier et al. observed a significantly steeper slope in the association between CRP and both visceral and subcutaneous adipose tissue in females compared to males (58), while Schorr et al. reported that visceral adipose tissue was more strongly associated with cardiometabolic risk markers in females, while intramyocellular lipids were more strongly associated with risk markers in males (59). Although our study did not assess fat distribution, our findings, interpreted in the context of these prior studies, may suggest potential sex-based variations in the association of adiposity with inflammation. This may have implications for clinicians deciding which patients to query about the possible downstream effects of inflammation such as heart disease and cancer. It is important to note that although the difference in the odds ratios in the sex-stratified analyses suggested that the association between body composition and inflammation may differ by sex, these comparisons were not derived from a formal sex × body composition interaction test. Given the overlapping confidence intervals, these findings should be interpreted as suggestive rather than conclusive and warrant confirmation in further research studies designed to evaluate potential sex-specific differences in the association of NWO with inflammation between males and females.

To our knowledge, this is the first nationally representative study of U.S. adults showing that NWO is associated with significantly higher odds of inflammation as measured by elevated hs-CRP levels, a widely used and clinically relevant biomarker of inflammation. This finding is supported by a recent systematic review of studies on NWO and inflammatory markers which found that NWO was associated with high levels of CRP and IL6 (60). For example, a prior study by De Lorenzo et al., included in the aforementioned systematic review, reported that having NWO was associated with significantly higher levels of interleukin and TNF-alpha (61). Although individuals with a normal BMI are typically considered as lower risk for chronic and cardiometabolic conditions, our findings indicate that those with a high BF% have significantly increased odds of systemic inflammation, which evidence shows plays an important role in the development and progression of multiple chronic diseases, including hypertension, atherosclerosis, coronary artery disease, type 2 diabetes, and cancer (1–15).

Our study contributes to a growing body of literature showing that excess body fat, even at a normal BMI, is associated with increased cardiometabolic risk (24, 30–38). Routine assessment of body composition could help identify individuals with NWO who may otherwise be missed by BMI-based screening alone. The recent 2025 statement from the American College of Cardiology (ACC) on inflammation and cardiovascular disease recommends hs-CRP measurement as part of CVD primary prevention, reinforcing the clinical relevance of inflammation as a modifiable risk factor (62). Our findings underscore the importance of identifying inflammation in individuals with normal BMI but excess adiposity and support calls to improve how body composition is assessed in clinical practice. While DXA is the gold standard, its cost and use of ionizing radiation limit its routine use (49, 50). Bioelectrical impedance analysis (BIA) is a low-cost alternative that may be more feasible for widespread implementation in primary care (63, 64). As such technologies become more accessible, incorporating BF% assessment into routine health screenings could enhance early identification of high-risk individuals and support more targeted prevention strategies, including early lifestyle interventions to reduce inflammation and cardiometabolic risk.

This study has several strengths, including the use of a nationally representative sample of U.S. adults, with data on BF% measured using DXA (a gold standard for body composition assessment) and inflammation measured using hs-CRP, a clinically relevant and widely used biomarker. Our categorization of individuals into four groups based on BMI and BF% enabled us to assess the joint association of these metrics as combined predictors of the odds of having systemic inflammation, as well as to identify and quantify associations with inflammation across distinct BMI-BF% phenotypes. Given the cross-sectional nature of the study, it is important to emphasize that these findings are associations rather than causal effects. While BMI and BF% were modeled as a composite variable, preventing direct comparisons between their individual associations with inflammation, our findings clearly demonstrate that BF% adds clinically meaningful information beyond BMI alone, thus underscoring the value of including BF% alongside BMI to more accurately assess the likelihood of inflammation and subsequent cardiometabolic and chronic disease risk in clinical settings.

Limitations of our study include its cross-sectional design, which prevents inference about causality or temporality, and reliance on a single hs-CRP measurement, which may not reflect long-term inflammatory status, as hs-CRP levels can fluctuate and may spike due to acute infections or injuries (65, 66). However, the results of a sensitivity analysis excluding individuals with hs-CRP levels > 10 mg/L confirmed the robustness of the main findings. Another limitation of this study is that the sample was restricted to adults aged 18–59 years due to NHANES DXA eligibility criteria; therefore, the findings apply only to non-elderly adults. We recommend that future research examine the association between NWO and inflammation in older adults as data on body composition becomes available, since the relationship may differ or be even more pronounced with aging, for example, in the context of sarcopenic obesity. Additionally, NHANES DXA eligibility criteria excluded those with self-reported weight > 450 pounds or height > 6 feet 5 inches, meaning that extremely high-BMI individuals were not represented. This could slightly bias the “elevated BMI and high BF%” group toward less extreme obesity. Although individuals exceeding these thresholds represent a very small fraction of the U.S. population, it is important to note that their inflammation levels might be even higher. In addition, although models adjusted for age and race/ethnicity, other potential confounders such as lifestyle behaviors and comorbidities were not included, as these variables may act as mediators on the pathway between body composition and inflammation. Therefore, the reported associations reflect relationships between BMI- and BF%-defined body composition profiles and inflammation as they exist in the general U.S. adult population, rather than associations independent of these factors. Thus, the potential for residual confounding cannot be excluded.

In summary, NWO is associated with markedly elevated odds of inflammation in U.S. adults, although causality cannot be inferred due to the cross-sectional design of the study. Compared with individuals who had both a normal BMI and a normal BF%, those with NWO had significantly higher odds of inflammation, while those with an elevated BMI but a normal BF% did not exhibit increased odds in the overall study population. These findings highlight the limitations of relying on BMI as a standalone measure of body composition and underscore the importance of incorporating BF% as part of a more comprehensive assessment to better evaluate chronic disease risk in the clinical setting. We recommend that clinicians interpret BMI cautiously and consider body fat percentage assessment, when feasible, to better identify patients at increased cardiometabolic and inflammatory risk.

## Data Availability

Publicly available datasets were analyzed in this study. This data can be found here: https://wwwn.cdc.gov/nchs/nhanes/default.aspx.
