# Supplementary material for: More evidence of the health risks of normal weight obesity: the association with systemic inflammation
Source: Front Med (Lausanne). 2025 Nov 18;12:1695935. doi: 10.3389/fmed.2025.1695935 (PMC12670252; doi:10.3389/fmed.2025.1695935)
Supplement: Supplementary file 1 [file Table_1.docx]

**Appendix Table A1.** Sensitivity Analysis Excluding Adults with hs-CRP Levels >10mg/L: Association Between Body Composition and Odds of Elevated hs-CRP Levels Among U.S. Adults Aged 18–59 Years: Unadjusted and Adjusted Odds Ratios from Survey-Weighted Logistic Regression, Overall and Stratified by Sex, NHANES 2017–2018.

| Body Composition Group | Unadjusted OR (95% CI) | P-Value | Adjusted OR* (95% CI) | P-Value |
| --- | --- | --- | --- | --- |
|  | | | |  |
| Overall Study Population | | | |  |
| Reference Group: Normal BMI with Normal BF% | 1.00 | - | 1.00 | - |
| Normal BMI with High BF% (Normal Weight Obesity) | 3.61 (1.89, 6.88) | 0.0021 | 3.43 (1.78, 6.61) | 0.0104 |
| Elevated BMI with Normal BF% | 1.26 (0.75, 2.11) | 0.4015 | 1.58 (0.98, 2.55) | 0.1121 |
| Elevated BMI with High BF% | 6.77 (4.13, 11.1) | <0.0001 | 7.14 (4.38, 11.6) | 0.0002 |
|  | | | |  |
| Males | | | |  |
| Reference Group: Normal BMI with Normal BF% | 1.00 | - | 1.00 | - |
| Normal BMI with High BF% (NWO) | 6.64 (2.13, 20.7) | 0.0068 | 6.33 (2.20, 18.2) | 0.0111 |
| Elevated BMI with Normal BF% | 1.28 (0.53, 3.11) | 0.5936 | 1.22 (0.53, 2.80) | 0.6617 |
| Elevated BMI with High BF% | 6.42 (3.48, 11.9) | <0.0001 | 5.96 (3.31, 10.7) | 0.0006 |
|  | | | |  |
| Females | | | |  |
| Reference Group: Normal BMI with Normal BF% | 1.00 | - | 1.00 | - |
| Normal BMI with High BF% (NWO) | 2.48 (1.09, 5.66) | 0.0518 | 2.46 (1.07, 5.64) | 0.0716 |
| Elevated BMI with Normal BF% | 2.23 (1.06, 4.68) | 0.0551 | 2.24 (1.08, 4.62) | 0.0661 |
| Elevated BMI with High BF% | 8.00 (4.17, 15.3) | <0.0001 | 7.93 (4.04, 15.5) | 0.0005 |

Individuals with BMI <18.5 kg/m² were excluded.

*Models adjusted for age and race/ethnicity.

P-values are based on design-adjusted t-distributions; minor differences from 95% CIs reflect survey-weighted estimation. Some sex-stratified subgroups (e.g., males with normal weight obesity and females with elevated BMI and normal BF%) had small unweighted sample sizes; therefore, the precision of these estimates (as reflected in their confidence intervals) should be interpreted with caution.
